# Complete Genome Sequence of *Bacillus subtilis* subsp. *subtilis* Strain ∆6

**DOI:** 10.1128/genomeA.00759-16

**Published:** 2016-07-28

**Authors:** Daniel R. Reuß, Andrea Thürmer, Rolf Daniel, Wim J. Quax, Jörg Stülke

**Affiliations:** a Department of General Microbiology, Georg-August-University Göttingen, Göttingen, Germany; b Department of Genomics and Applied Microbiology and Göttingen Genomics Laboratory, Georg-August-University Göttingen, Göttingen, Germany; c Groningen Research Institute of Pharmacy, University of Groningen, Groningen, The Netherlands

## Abstract

*Bacillus subtilis* ∆6 is a genome-reduced strain that was cured from six prophages and AT-rich islands. This strain is of great interest for biotechnological applications. Here, we announce the full-genome sequence of this strain. Interestingly, the conjugative element ICE*Bs*1 has most likely undergone self-excision in *B. subtilis* ∆6.

## GENOME ANNOUNCEMENT

*Bacillus subtilis* ∆6 is a derivative of the laboratory wild-type strain *B. subtilis* 168, which was cured from six prophages and AT-rich islands. For this purpose, the prophages SPβ and PBSX, the prophage-like elements prophage 1, prophage 3, and *skin*, as well as the polyketide synthesis operon *pks* were deleted. Interestingly, this genome reduction by 7.7% did not have a major impact on physiology, metabolic flux patterns, or genetic competence (1).

*B. subtilis* ∆6 is a promising starting point for further genome reduction. Moreover, it can serve as a chassis strain in the context of biotechnological applications, that is, highly efficient protein secretion and vitamin production (2–4). Indeed, *B. subtilis* ∆6 has recently been used to obtain a total genome reduction of 13.6% (5). For a better understanding with respect to future projects, we have sequenced the genome of *B. subtilis* ∆6. The chromosomal DNA was isolated from a stationary phase culture using a commercially available kit (peqGOLD Bacterial DNA Kit, VWR International GmbH). We obtained 6.63 million reads from an Illumina 75-bp single-read run and mapped them to the *B. subtilis* 168 genome (GenBank accession number NC_000964) (6) using the Geneious Read Mapper (Geneious version 9.0.5 software, Biomatters, Ltd.) (7). The alignment showed a 118-fold average coverage and a 99.5% pairwise identity to the reference genome of *B. subtilis* 168. The insertion and the correct sequence of the chloramphenicol resistance gene at the *pks* operon locus were verified by a standard PCR. The final genome sequence of *B. subtilis* ∆6 has a length of 3,876,919 bp.

We identified 28 variations (single-nucleotide polymorphism, deletion, insertion, and substitution) with a minimal coverage of 25× and a minimum variant frequency of 0.8. Four of these mutations have an effect on the amino acids sequence of the encoded protein (*carA*, *yobM*, *ywbD*, and *walH*), whereas four mutations are silent (*yczC*, *yjnA*, *glcF*, and *amyX*). The remaining 20 variants are located in intergenic and RNA-encoding regions. All variations can be requested from the corresponding author. In addition, we could confirm the presence of all six deletions performed by Westers et al. (1). Interestingly, *B. subtilis* ∆6 contains a seventh large deletion of 20.5 kb (25 genes; genome position: 529,422 to 549,925 bp). This deletion corresponds to the mobile genetic element ICE*Bs*1 (8), which likely has undergone self-excision, as it has been reported for other *B. subtilis* strains (9). Taken together, *B. subtilis* ∆6 is lacking 376 genes at seven different locations covering 8.03% of the reference genome of *B. subtilis* 168. These deletions increased the GC content from 43.5% to 43.9%.

### Nucleotide sequence accession number.

The genome sequence of *B. subtilis* subsp. *subtilis* strain ∆6 is deposited in GenBank under the accession number CP015975.
